# Starr-Edwards Valve Explantation After 40 Years

**DOI:** 10.1016/j.atssr.2026.02.031

**Published:** 2026-03-18

**Authors:** Joshua G. Crane, Mark S. Slaughter

**Affiliations:** 1 Department of Cardiovascular and Thoracic Surgery, University of Louisville, Louisville, Kentucky

**Figure 1:**
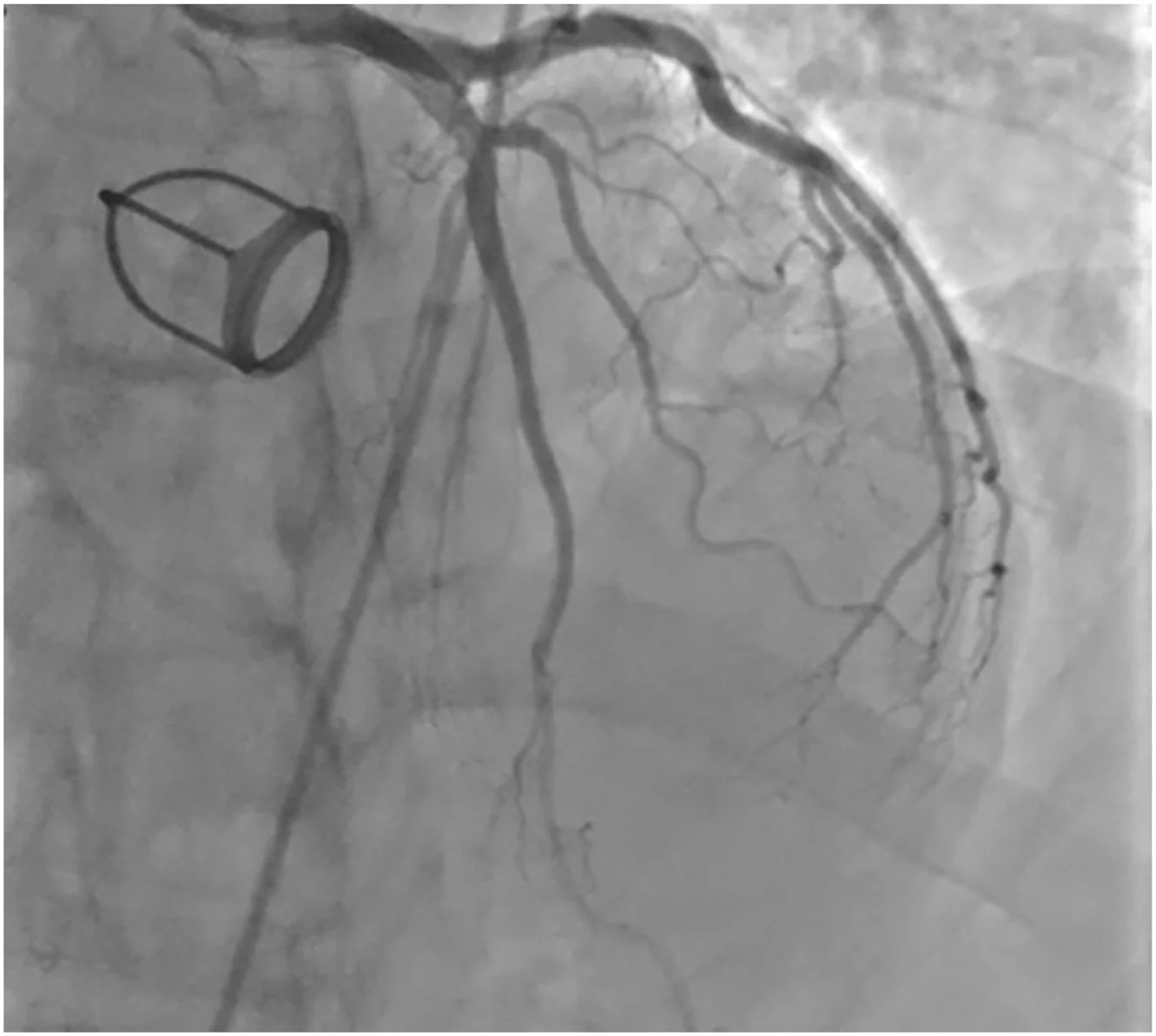

**Figure 2:**
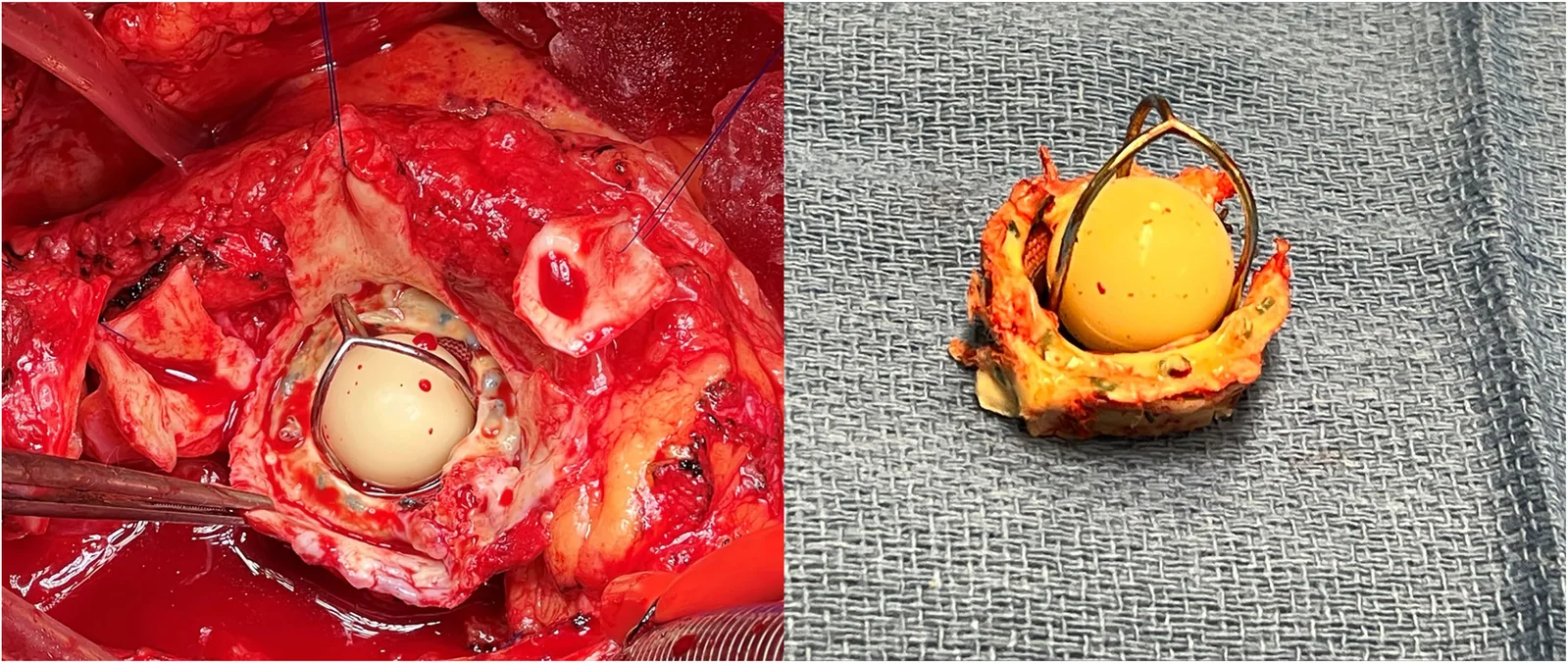

A 61-year-old patient was referred for an enlarging ascending and aortic root aneurysm, which measured 6.8 cm (Supplemental Video 1). At age 21, the patient underwent an aortic valve replacement with a Starr-Edwards ball-in-cage valve (Figure 1) at another hospital for aortic valve endocarditis. The patient had been maintained on warfarin and done well for 40 years, with no evidence of valve dysfunction (Supplemental Videos 2 and 3). Now, the patient required aortic valve and root replacement with explantation of the Starr-Edwards ball-in-cage valve. The valve explant and root replacement was completed using a KONECT composite graft (Edwards Lifesciences). In vivo, the ball-in-cage valve remained intact and viable after 40 years (Figure 2).

Initial efforts to build an artificial heart “one valve at a time” led to development of a ball-in-cage valve design. The first Starr-Edwards valve was implanted in the mitral position in 1960.^1^ After its widespread implantation in aortic and mitral positions, the Starr-Edwards valve showed resiliency beyond 30 years.^2^ Since that time, valve technology has advanced tremendously, allowing implantation of tissue valves using small-caliber transcatheter devices. This image highlights the resilience of historic valve technology design and is a reminder of the tremendous advances and success of current catheter-based technology.
